# Clonal phylogenies inferred from bulk, single cell, and spatial transcriptomic analysis of epithelial cancers

**DOI:** 10.1371/journal.pone.0316475

**Published:** 2025-01-03

**Authors:** Andrew Erickson, Sandy Figiel, Timothy Rajakumar, Srinivasa Rao, Wencheng Yin, Dimitrios Doultsinos, Anette Magnussen, Reema Singh, Ninu Poulose, Richard J. Bryant, Olivier Cussenot, Freddie C. Hamdy, Dan Woodcock, Ian G. Mills, Alastair D. Lamb

**Affiliations:** 1 Nuffield Department of Surgical Sciences, University of Oxford, Oxford, United Kingdom; 2 Research Program in Systems Oncology, University of Helsinki, Helsinki, Finland; 3 Department of Urology, Oxford University Hospitals NHS Foundation Trust, Oxford, United Kingdom; PSTU: Patuakhali Science and Technology University, BANGLADESH

## Abstract

Epithelial cancers are typically heterogeneous with primary prostate cancer being a typical example of histological and genomic variation. Prior studies of primary prostate cancer tumour genetics revealed extensive inter and intra-patient genomic tumour heterogeneity. Recent advances in machine learning have enabled the inference of ground-truth genomic single-nucleotide and copy number variant status from transcript data. While these inferred SNV and CNV states can be used to resolve clonal phylogenies, however, it is still unknown how faithfully transcript-based tumour phylogenies reconstruct ground truth DNA-based tumour phylogenies. We sought to study the accuracy of inferred-transcript to recapitulate DNA-based tumour phylogenies. We first performed in-silico comparisons of inferred and directly resolved SNV and CNV status, from single cancer cells, from three different cell lines. We found that inferred SNV phylogenies accurately recapitulate DNA phylogenies (entanglement = 0.097). We observed similar results in iCNV and CNV based phylogenies (entanglement = 0.11). Analysis of published prostate cancer DNA phylogenies and inferred CNV, SNV and transcript based phylogenies demonstrated phylogenetic concordance. Finally, a comparison of pseudo-bulked spatial transcriptomic data to adjacent sections with WGS data also demonstrated recapitulation of ground truth (entanglement = 0.35). These results suggest that transcript-based inferred phylogenies recapitulate conventional genomic phylogenies. Further work will need to be done to increase accuracy, genomic, and spatial resolution.

## Introduction

It is generally accepted that cancers develop and evolve by adaptive genetic and molecular changes over time [1–3]. Sequential selection from this process of evolution leads to clones and subclones with altered phenotype leading to more aggressive behaviour. Ultimately, these phenotypic changes lead to metastatic spread and drug resistance, which is responsible for the majority of cancer-related deaths [4].

It is necessary to distinguish accurately tumour heterogeneity and determine clonal evolution by identifying the clonal source of metastatic disease. This not only has an impact on the understanding of tumour progression but the relationship between clonal composition and the index lesion is also important and clinically relevant for both molecular diagnostics and focal therapy [5–8]. Indeed, it would help and support treatment decision-making by using new markers to determine whether cells are indicative of aggressive disease or to predict sensitivity to treatment.

One of the challenges to understand the tumour heterogeneity is that the origin of mutations occurring in cancer can be hereditary or somatic. Although identification of inherited mutations is relatively straightforward, these are only responsible for 5 to 10% of all cancer [9–11]. By contrast, post-developmental somatic genetic alterations are usually only present in a small fraction of clonally-expanding cells but constitute the most common cause of cancer [12]. To identify these somatic mutations *in situ*, techniques such as laser capture microdissection have been employed, but this requires pre-knowledge to isolate a specific cell type or region of interest from a tissue section [13] and so limits the ability to undertake a *de novo* spatial clonal analysis. Recently, these limitations have been overcome by spatial transcriptomics, which allows the analysis of gene expression profiles in a tissue sample while preserving spatial tissue architecture. This approach captures transcripts *in situ*, with sequencing of barcoded reads carried out *ex situ* and then mapped back to the cells of origin [14, 15]. This cutting-edge technology permits visualisation and in-depth analysis of intra-tumoural heterogeneity and could permit spatial analysis of clonal evolution.

Clonal evolution and, more precisely, the relationship between clones and subclones is often represented and visualised by phylogenetic trees [16, 17]. These phylogenetic trees have been used mainly in recent years to study data derived from DNA sequencing [17]. However, to use spatial transcriptomics to study clonal evolution, it is necessary to know whether RNA can also be used to determine clonal phylogenetic hierarchies. In this meta-analysis, we investigate the correlation between DNA sequencing data and RNA sequencing data using phylogenies derived from inferred single-nucleotide variants (SNV) and copy-number variants (CNV) in order to determine whether transcriptome-derived phylogenies can accurately reflect genome-based phylogenies.

## Materials and methods

### Data acquisition

In order to benchmark and validate methods to generate phylogenies derived from inferred single-nucleotide variants and copy-number variants, we reviewed the literature and found a recent publication which simultaneously extracted both DNA and RNA, from the same exact single tumour cells, and performed whole genome and whole transcriptome sequencing [18]. These public datasets contained data from 38 single cells that had been subject to simultaneous WGS and RNAseq using the SIDR methodology. Han et al describe a quality control process to determine which cells were satisfactorily sequenced for downstream analysis, leaving a total of 30 paired samples that passed all qc metrics [18].

Next, we reviewed the literature for publications and available data from patients with prostate cancer, who had both conventional bulk DNA and RNA sequencing applied to the same specimen, and from patients that had three or more total specimens. We identified patient A21 [19, 20], patient 498 [21]. For further validation and comparison, WGS and RNA-microarray data were obtained from cases 6, 7 and 8 from Cooper et al. [22].

Lastly, we obtained paired WGS sequencing data and paired Spatial Transcriptomics data from the n = 12 regions from a single patient in a recent publication [23].

### Analysis of single cell data

#### Quality control of single-cell whole genome sequencing data

Only 38 paired cells were available with both scWGS and scRNAseq [18]. After removing the individual cells that failed either scWGS or scRNAseq QC left only 30 in common.

#### DNA sequencing preprocessing of single-cell whole genome sequencing data

Paired end sequencing data was aligned against the GRCh38 reference genome with the Burrow-Wheeler Aligner (0.7.17).

#### iSNV calling from single-cell whole genome sequencing data

WGS variants were called using a pipeline broadly based on the GATK best practice Germline short variant discovery (SNPs + Indels) workflow using Picard (2.23.0) and GATK (4.1.7.0). This consisted of pre-processing the raw alignment to mark duplicate reads and perform base recalibration. Raw variants were called using GATK HaplotypeCaller in GVCF mode followed by GATK GenotypeGVCFs. Finally the raw variants were filtered to generate a downstream analysis ready cell by variant dataset.

The processed variants were converted to an Identity by State matrix, clustered and converted to dendrogram format in R using the SNPrelate package [24, 25].

#### gCNV calling from single-cell whole genome sequencing data

After preprocessing and QCing, n = 30 cells remained, and were then analyzed by Gingko [26]. BAM files were converted to.BED files using bamToBed in BedTools. We utilized a variable bin size of 50 kb, with 101 bp reads [18]. The clustering of CNV’s was performed using ward linkage and Euclidean distance as the distance metric. Copy-Number tree results were downloaded in Newick format for further downstream analysis.

#### RNA sequencing preprocessing of single-cell whole transcriptome sequencing data

Paired end sequencing data was aligned against the GRCh38 reference genome with STAR (2.7.3a) with per-sample 2-pass mapping and annotation with comprehensive gene annotation data from GENCODE GRCh38. Gene counts per cell were tabulated from aligned data using the featureCounts function from the Subread (1.6.4) package.

#### iSNV calling from single-cell whole transcriptome sequencing data

iSNV calling from RNAseq data was performed according to the pipeline outlined by Zhou et al and based on GATK best practices [27]. The STAR aligned data underwent sorting, annotation with read group information, deduplication, SplitNCigarReads, realignment, and base recalibration, before variant calling with GATK (3.8.0) HaplotypeCaller. Raw iSNVs were processed by DENDRO to calculate a genetic divergence matrix between cells and to generate a phylogeny using hierarchical clustering (ward.D method).

#### iCNV calling from single-cell whole transcriptome sequencing data

Data were analyzed using R version 4.0.1, and inferCNV (version 1.4.0) [28]. A merged file from the previously described pre-processing steps, containing feature counts for each cell, as well as a gene position file, and an annotation file were generated for input to inferCNV. An inferCNV object was created with no defined reference group. After creation of the InferCNV object, inferCNV was ran with the following parameters: cutoff = 0.1, cluster_by_groups = FALSE, denoise = TRUE, HMM = TRUE.

#### Comparison of dendrograms from single-cells

For comparison of dendrograms created by WGS-CNVs (Gingko) and inferred CNV’s from RNA (InferCNV), the clust2.newick and infercnv.21_denoised.observations_dendrogram.txt files were imported into R and analyzed with packages dendextend and phylogram.

### Analysis of transcript derived phylogenies

RNA counts were analzyed, by comparing individual gene count values to the median (MED) and standard deviation (SD) values of global RNA count values per sample: if the count value was less than MED-SD, then it was assigned a value of -1, else if the count value was greater than MED+SD, then it was assigned a value of +1, else it was assigned 0. The resultant values from each sample or cell were converted into a phydat object using *phangorn*’s function phyDat(), with the parameters type = "USER", levels = c(’-1’, ’0’, ’1’). Pairwise distances between cells or tissue samples were calculated using the *phangorn* dist.ml() function with previously described phyDat() object as input. UPGMA clustering was applied using the *phangorn* upgma() function and converted to a dendrogram using the *dendextend* function as.dendrogram().

### Analysis of spatial transcriptomics data

#### CNV calling from spatial transcriptomics data

Data were analyzed as previously described [29] with the following exceptions. Original 1k array Spatial Transcriptomics data were obtained. As gCNV comparison data were from whole sections, all ST count data were ‘pseudo-bulked’ within sections, resulting in 12 pseudobulked count matrices for analyses. InferCNV was ran using standard parameters with no reference set. The resultant *infercnv*.*observations_dendrogram*.*txt* dendrogram was used for downstream tanglegram analysis.

#### Comparison of dendrograms from WGS and ST

The original outputs for CNV calling from Berglund et al., were not available, and the ReadDepth package used to generate the calls has since been deprecated by the author [30]. Thus, we ran a new pipeline using the WGS data from Berglund et al [23]. FASTQ files were obtained and aligned to HG38. Battenberg CNV analyses [31] were performed using the matched reference blood FASTQ data as the reference.

#### Copy number calling with Battenberg

The Battenberg package (v2.2.10) was used to determine copy number, and estimate tumour purity and ploidy from WGS data. Impute2 (v2.3.0) was used with GRCh38 loci for phasing germline heterozygous SNPs. The Battenberg pipeline was run with the following parameters: segmentation_gamma = 10, phasing_gamma = 10, platform_gamma = 1, min_ploidy = 1.6, max_ploidy = 4.8, min_rho = 0.13, max_rho = 1.02.

The *recal_subclones*.*txt* text files were downloaded for each of the 12 prostate tissues, and processed through a custom pipeline as follows. Battenberg CNV segments were binned into 1200 bp segments and aligned, generating n = 2439447 bins across the genome. CN amplifications and deletions were called at thresholded values of -1.5 and 2.5 respectively. Next, the processed bins from all samples were merged to create a CN bin matrix. CN calls for segments that were shared for all samples were dropped, resulting in a final matrix containing n = 28 discordant CN calls.

This CN matrix was then used similarly as described by Berglund et al., with the R package *pvclust*, and n = 1000 bootstraps. The structure of the cluster was converted to a dendrogram using the R package *dendrogram* for comparison to the inferCNV dendrogram via a tanglegram using the *dendextend* package (step2side).

## Results

### Transcriptome and genome derived clonal phylogenies from single cancer cells

In order to benchmark performance of transcriptome-derived phylogenies, we first identified an individual cancer cell dataset with simultaneously isolated DNA and RNA (SIDR) from single cells [18]. The SIDR approach resulted in paired DNA and RNA nucleic acid extractions from isolated single cells of three different cancer cell lines: HCC827, MCF7 and SKBR3 [18]. They then performed whole-genome sequencing (WGS) and RNA-sequencing on the extracted nucleic acids [18]. Given the cell purity, we hypothesized that WGS and RNA sequencing data from these individual cancer cells could be analyzed in an “in-silico” experiment to benchmark performance of transcriptome and genome-derived phylogenies.

**Fig 1:**
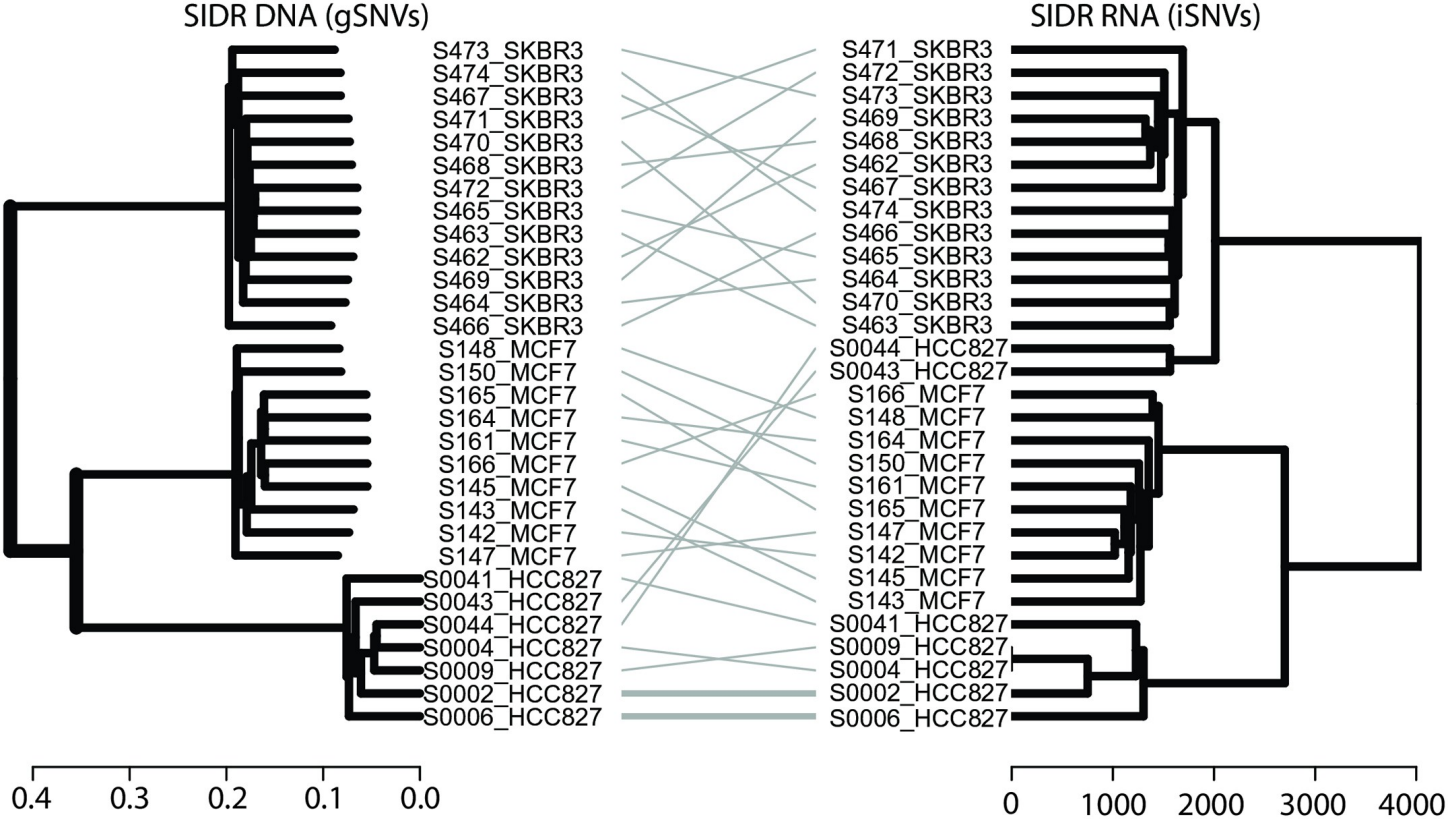
Comparison of in-silico clonal phylogenies from single tumour cells with co-isolated DNA and RNA (Han et al., Genome Res 2018). Dendrograms constructed from clustering of transcript-based inferred single-nucleotide variants (DENDRO) and ground truth DNA-based single-nucleotide variant calls (GATK) and compared by tanglegram. Colours correspond to individual cell lines (yellow: SKBR3, green: HCC827, and light blue: MCF7). Entanglement of the phylograms was 0.097 (an entanglement value of 1 corresponds with full entanglement of two phylograms, whereas an entanglement value of 0 corresponds with no entanglement).

**Fig 2:**
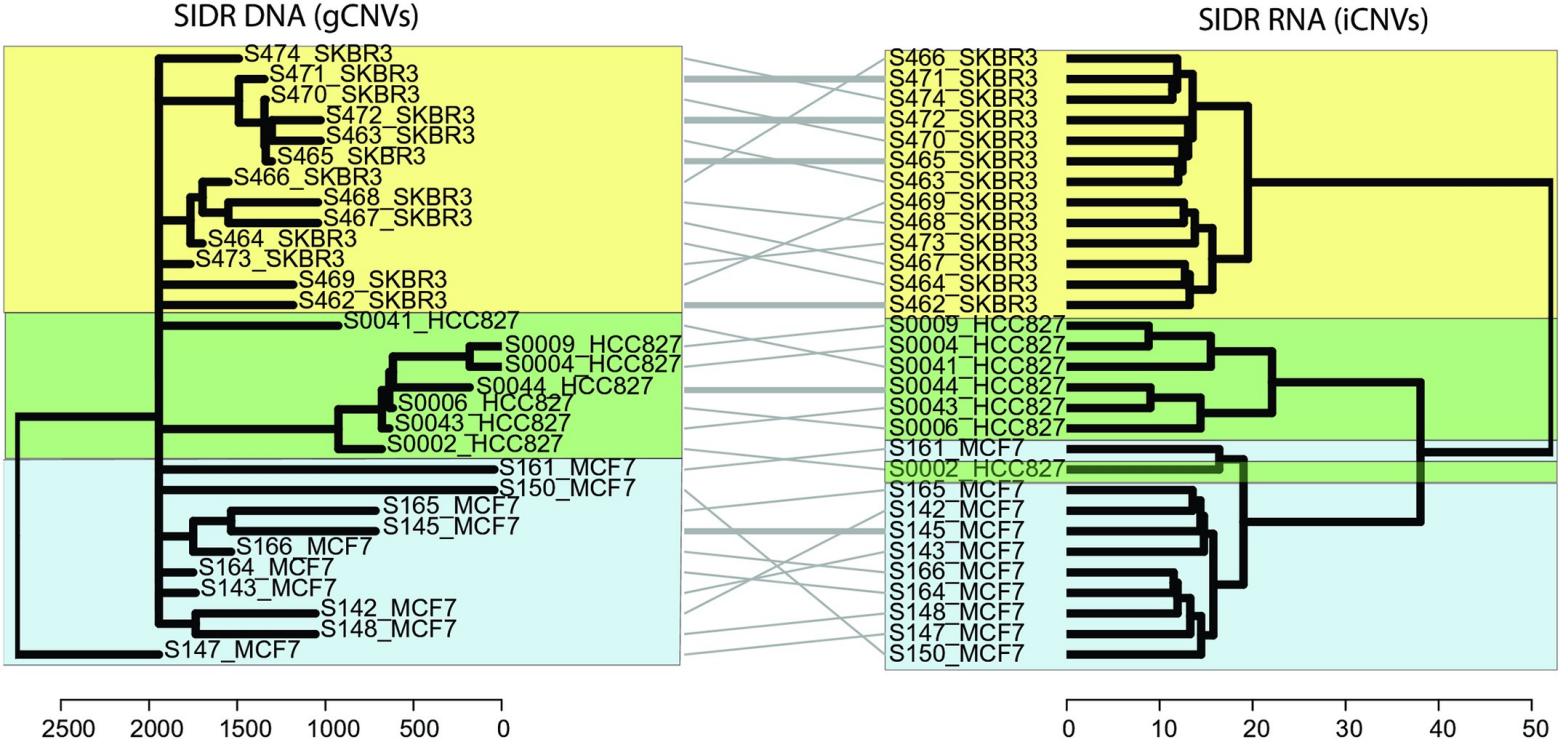
Comparison of in-silico clonal phylogenies from single tumour cells with co-isolated DNA and RNA (Han et al., Genome Res 2018). Dendrograms constructed from clustering of transcript-based inferred copy-number variants (inferCNV) and ground truth DNA-based copy number variant calls (WGS-Ginkgo) and compared by tanglegram. Colours correspond to individual cell lines (yellow: SKBR3, green: HCC827, and light blue: MCF7). Entanglement of the phylograms was 0.11 (an entanglement value of 1 corresponds with full entanglement of two phylograms, whereas an entanglement value of 0 corresponds with no entanglement). As adapted from Erickson et al., Nature, 2022, Extended Data Fig 1a.

We performed secondary analyses of the published, publicly available DNA and RNA sequencing data from Han et al [18]. After quality control [18], we identified a total of 30 cells that had both sufficient quality DNA and RNA sequencing data, resulting in a dataset of a total of 10 MCF7 cells, 7 HCC827 cells, and 13 SKBR3 cells for analysis. We performed genomic SNV (gSNV) and inferred RNA-based SNV (iSNV) analyses from all cells, derived dendrograms, and performed tanglegram analysis to compare gSNV and iSNV dendrograms. In analysis of gSNVs and iSNVs, we observed a high concordance of transcriptome and genomic phylogenies (Fig 1, entanglement = 0.097). Next, we performed genomic CNV (gCNV) and inferred RNA-based CNV (iCNV) analyses from all cells, derived dendrograms, and performed tanglegram analysis to compare gCNV and iCNV dendrograms. In analysis of gCNVs and iCNVs, we also observed a high concordance of transcriptome and genomic phylogenies (Fig 2, entanglement = 0.11). We therefore concluded that RNA-derived inference of genomic SNVs and CNVs in three purified single cell populations generated strong phylogenetic concordance.

### Transcriptome and genome derived clonal phylogenies from bulk prostate cancer sequencing

Having established high *in-silico* concordance of transcriptome and genome-derived phylogenies, we then sought to study prostate cancer sequencing data from patients with paired DNA and RNA extracted from the same tumours. Gundem and colleagues reported WGS data from 55 disseminated tumour samples, from 10 patients that underwent rapid-autopsy after death due to prostate cancer [19]. A subset of n = 7 tumour specimens from patient A21 also underwent RNA-sequencing [20].

**Fig 3:**
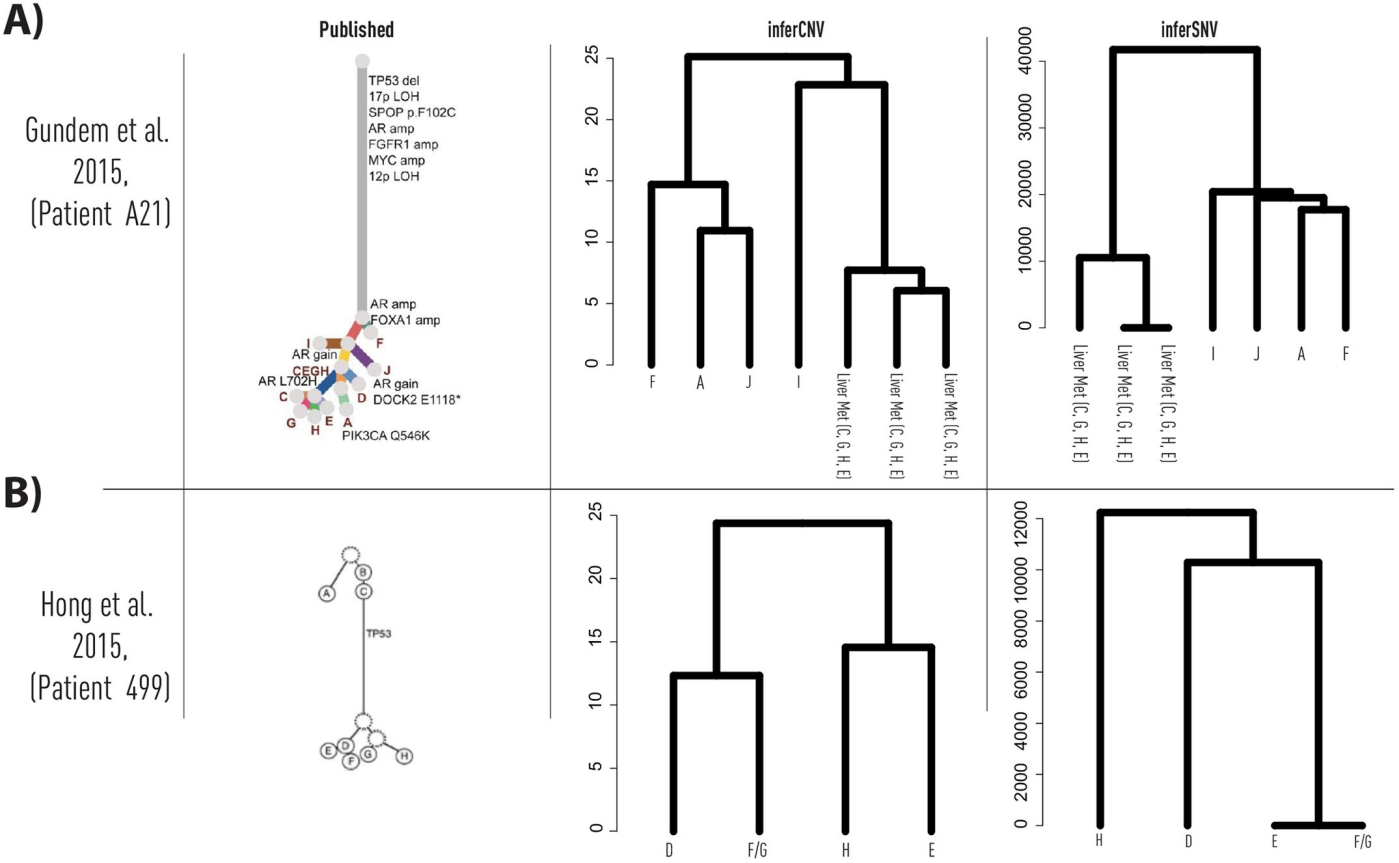
Comparison of published DNA-based prostate cancer clonal phylogenies and transcript-based inferred single-nucleotide and copy-number variant derived dendrograms. a) Phylogeny from patient A21, as published and reproduced from Gundem et al., Nature, 2015. Transcript data were available only for a subset of specimens. b, Phylogeny from patient 498, as published and reproduced from Hong et al., Nat. Comms, 2015. Transcript data available for a subset of specimens. inferCNV-based clonal phylogenies adapted from Erickson et al., Nature, 2022, Extended Data Fig 1b.

We performed secondary analyses of RNA sequencing data from Bova et al. and obtained iSNV and iCNV calls. From the iSNV and iCNV calls, we separately performed phylogenetic analyses through hierarchical clustering, resulting in iSNV and iCNV derived dendrograms (Fig 3a). In both iSNV and iCNV analyses, liver metastases (C, G, H, E) clustered together. In both iSNV and iCNV analyses, Clones F, A and J also clustered together. Clone I, clustered together with the liver metastases in iCNV analyses, but not in the iSNV analyses. Taken together, the iSNV and iCNV dendrograms reflect the manually assembled clonal phylogeny published by Gundem et al, [19].

Next, we analyzed data from patient 498, analyzed by Hong et al.[21]. This patient’s primary prostate cancer progressed to distant skeletal metastases, which then further re-seeded the prostatic bed. Of the n = 7 reported specimens, a total of n = 4 also underwent RNA sequencing. We performed secondary analyses of the RNA sequencing data and obtained iSNV and iCNVcalls. From the iSNV and iCNV calls, we separately performed phylogenetic analyses through hierarchical clustering, resulting in iSNV and iCNV derived dendrograms (Fig 3b). In contrast to the results from Gundem et al., both iSNV and iCNV presenting differing tree patterns as compared to one another.

**Fig 4:**
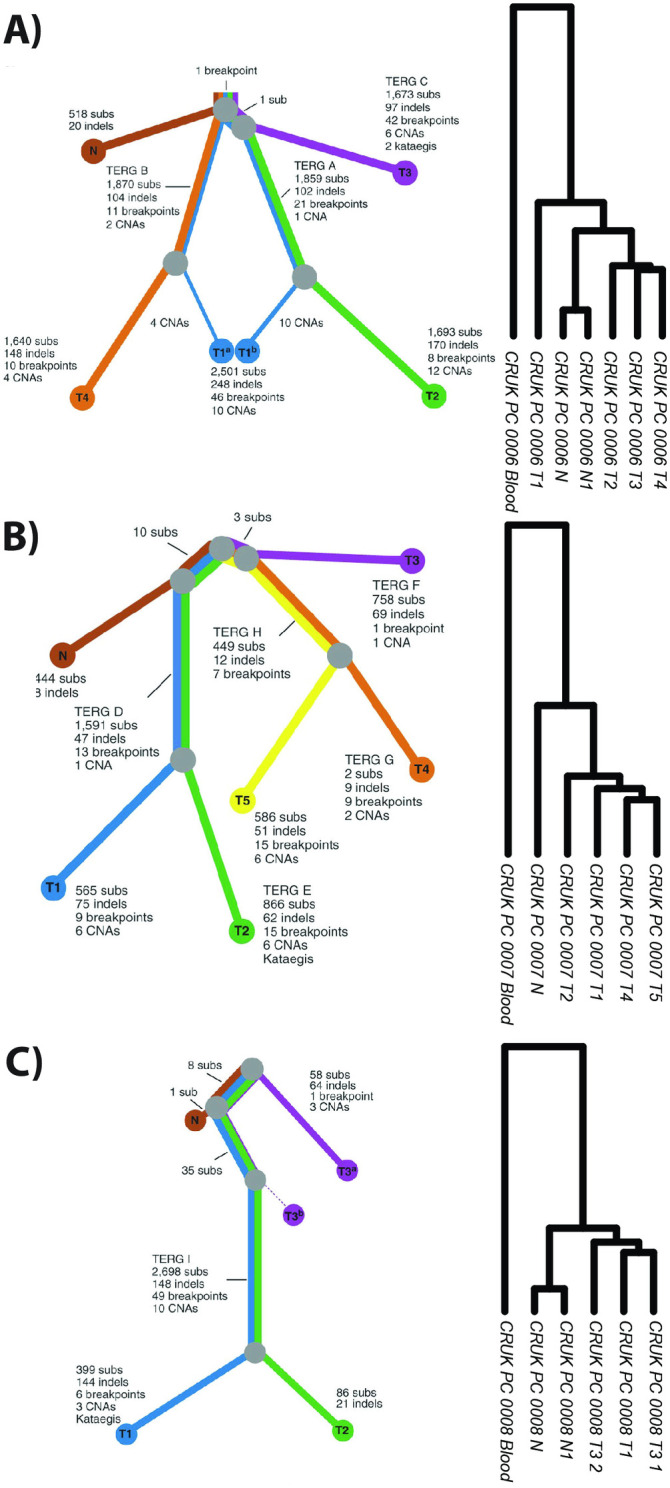
Comparison of published DNA-based (WGS) phylogenetic trees (left) as compared to novel RNA-based (RNA Microarray) phylogenies (right) from Cooper et al., 2015. A) Phylogenies from patient CRUK0006, B) Phylogenies from patient CRUK0007, C) Phylogenies from patient CRUK0008. RNA phylogenies include blood samples not presented in DNA-based phylogenetic trees.

We then analyzed data from primary prostate cancer cases 6, 7 and 8, analyzed by Cooper et al., who each underwent radical prostatectomy, from which multiple tissue punches of both normal and tumour regions were sampled [22]. The samples then underwent WGS, which were subsequently analyzed and tumour phylogenies were manually produced. From a subset of the same specimens, adjacent tissue sections were taken and subjected to RNA microarray analysis. Additionally, each patient had a blood sample taken, that also underwent RNA microarray analysis. Being microarray data, we were unable to derive iSNV and iCNVs. Therefore, we built a custom pipeline to analyze and cluster the RNA microarray data directly, to generate hierarchical clustering represented as a dendrogram. To benchmark this pipeline, we first compared gCNV and gSNV to SIDR data (S1 Fig) and observed entanglement values of 0.21 and 0.16 respectively. Having established this pipeline, we then applied it to the microarray data from Cooper et al to generate dendrograms. These dendrograms were then analyzed in comparison to the published WGS-based gDNA phylogenies (Fig 4). In all three patients, the blood specimen clustered separately from the prostate tumour and normal tissue specimens. In cases 7 and 8, the (multiple) normal tissue specimens clustered together and distinctly clustered separately from the tumours, whereas in case 6 the two normals clustered with T_2_, T_3_ and T_4_, separate from T_1_. Taken together, RNA-microarray derived dendrograms were able to recapitulate manually assembled WGS-derived gDNA phylogenies.

### Transcriptome and genome derived clonal phylogenies from bulk WGS and spatial transcriptomics from multi-region prostate cancer sequencing data

**Fig 5:**
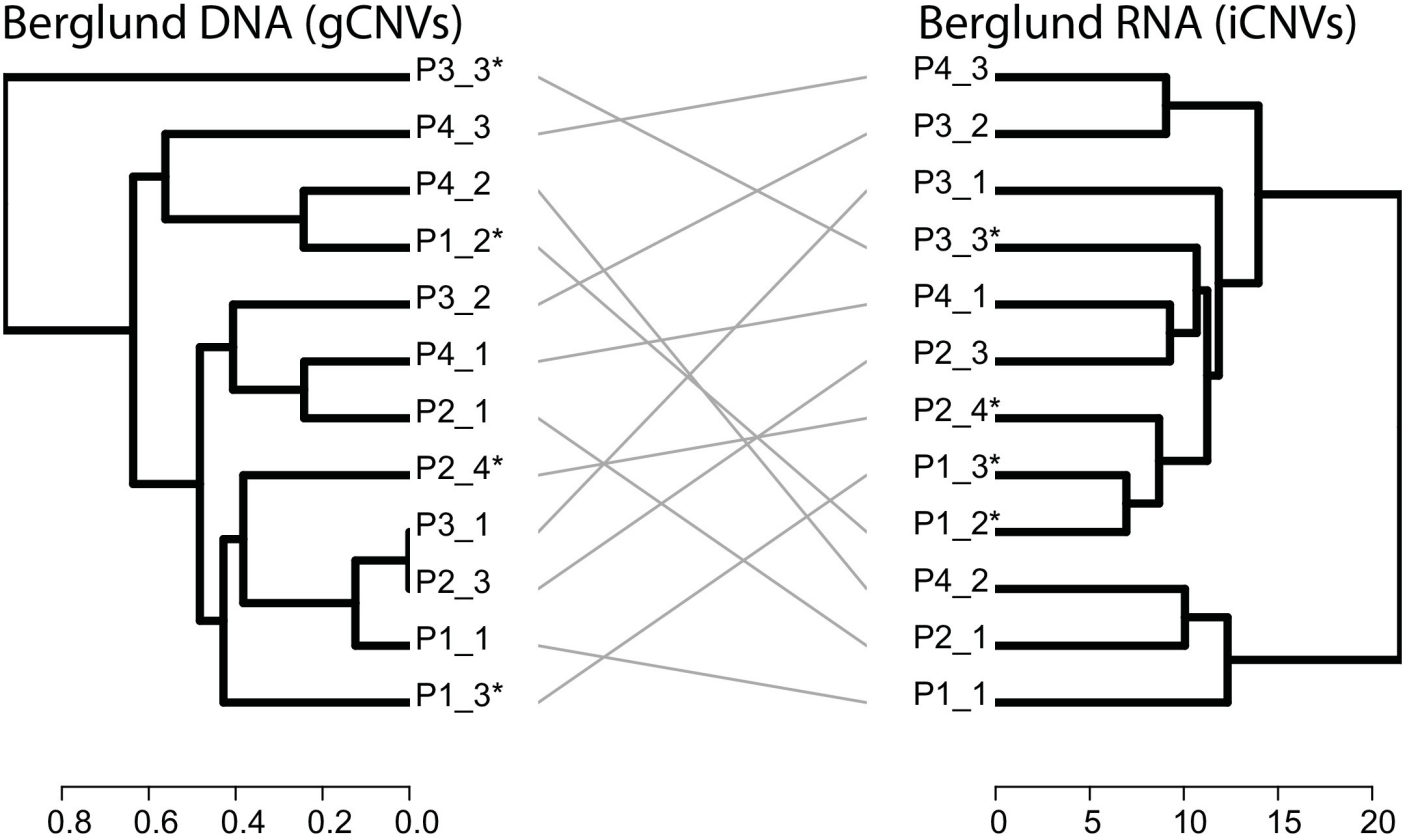
Comparison of DNA-based (WGS) phylogenetic trees (left) as compared to transcript-based inferCNV clonal phylogenies (right) from Berglund et al., 2018. DNA dendrogram constructed using patient-matched blood sample as a reference: such data were not available for inferCNV. Entanglement of the phylograms was 0.35 (an entanglement value of 1 corresponds with full entanglement of two phylograms, whereas an entanglement value of 0 corresponds with no entanglement). A label with the ending of * represents a section containing histologically detected cancer.

Next, we then sought to determine the ability of spatial transcriptome derived tumour phylogenies to recapitulate gDNA based phylogenies. Spatial transcriptomics generates transcriptome signal from poly-A captured short 3’ RNA sequences of up to 200 bp length, sufficient for hg38 alignment and, we deduced, sufficient to enable iCNV analysis. Berglund and colleagues performed spatial transcriptomics (ST) [15] on a total of n = 12 prostate tissue regions from a patient that underwent radical prostatectomy [23]. Of these sections, a total of n = 4 were detected to have prostate cancer. The authors also performed WGS on adjacent serial sections from each of these 12 tissue sections, as well as a matched blood specimen from the same patient. Given that WGS is not spatially resolved, we performed ‘pseudo-bulked’ iCNV analyses on ST data from all 12 sections, and generated a clonal phylogeny in the form of a dendrogram. We also performed gDNA CNV calling from each of the 12 sections to generate a clonal phylogeny which was represented as a dendrogram. We then compared the iCNV and gCNV derived dendrograms using a tanglegram and observed a degree of concordance consistent with the resolution of the data (Fig 5, entanglement = 0.35). Interestingly, three of the tumour regions (P2_4, P1_3, P1_2) clustered together in the iCNV analysis, whereas they were represented on different subclusters in the gCNV phylogeny, suggesting that the iCNV approach may have generated a more accurate clustering in this case.

## Discussion

Results from single-cancer cells demonstrate that transcriptome-derived iCNV and iSNV phylogenies are highly concordant with ground truth gDNA based phylogenies. In our in-silico analyses, the analysed data represent a highly selected and well controlled set of cells, with a 1:1 pairing of data resulting in extremely low entanglement values of the resultant tanglegrams. These results are in line with findings by Han et al., where they reported positive correlations for all three cell lines between gCNV and mRNA expression levels that were binned across the genome [18]. Our quantitative results in single-cells were supported by qualitative comparisons in prostate cancer cells where we did not have access to all ground truth data to enable a true like-to-like comparison.

There are limitations to consider in the construction of transcriptome-derived inferred phylogenies. First, the design and resolution of the genetic sequencing technologies can greatly affect the ‘resolved signal’. For example, only 2% of the entire genome is translated into proteins [32], and thus the genomic coverage of the transcriptome represents a sub-fraction of potential data for mapping tumour phylogenies. This is further compounded by variable coverage within transcripts themselves: many modern scRNAseq and spatial transcriptomics techniques, such as Chromium and Visium offered by 10x Genomics, perform polyA capture, resulting in sequencing of 75–300 bp near the end of transcripts. Further, for iSNV approaches [27, 33], the coverage of transcribed SNV loci can be extremely low being confined to the exome. Potential issues with iSNVs seem to be mitigated in iCNV approaches [34–36], which incorporate machine learning algorithms to bin genomically adjacent transcripts. Additionally transcriptional regulation programs [37–39] can affect transcription without any changes to copy-number status: these may result in false positives or negatives in iCNV analyses. Indeed, Han et al observed a discrepancy in Chromosome 3 gCNV calls and expression profiles [18]. Finally, one key factor affecting the ability of iCNV/iSNV (as well as gCNV and gSNV) approaches is use of well annotated references. All of the patient-derived WGS analyses in the data used in this publication had access to reference blood controls for calling gCNVs and gSNVs. Such data are not often taken or obtained for RNA sequencing, and thus are unavailable for iCNV and iSNV calling. This can also be further compounded by tissue or cell-of-origin transcriptional programs unrelated to copy-number alterations. Spatial transcriptomic data offers the opportunity to compensate for this through selection of histologically normal regions as control references.

As the tumour evolution community moves increasingly to single cell and spatial resolution, our ability to resolve clonal and subclonal tumour evolution patterns will greatly increase. Our results underscore the need for proper reference sets when calling iCNV and iSNV derived clonal phylogenies. These issues may be partly mitigated by next-generation iCNV and iSNV algorithms that incorporate both into combined iSNV+iCNV phylogenies [40]. Other approaches incorporating evolutionary game theory through mathematical models could aid in resolving clonal phylogenies [41]. Further work will also need to be done to identify and control for non copy-number alteration derived transcriptional regulation leading to further refinements in the ability of transcript-based clonal phylogenies to resolve ground truth.

## Conclusions

These results suggest that transcript-based inferred phylogenies recapitulate conventional genomic phylogenies. As the tumour evolution community moves increasingly to single cell and spatial resolution, our ability to resolve clonal and subclonal tumour evolution patterns will greatly increase. Further work will need to be done to increase accuracy, genomic, and spatial resolution.

## Supporting information

S1 Fig Comparison of in-silico clonal phylogenies from single tumour cells with co-isolated DNA and RNA (Han et al., Genome Res 2018). A) Dendrograms constructed from ground truth DNA-based copy number variant calls (WGS-Ginkgo) and direct transcripts (hierarchical clustering) and compared by tanglegram. Colours correspond to individual cell lines (yellow: SKBR3, green: HCC827, and light blue: MCF7). Entanglement of the phylograms was 0.21 (an entanglement value of 1 corresponds with full entanglement of two phylograms, whereas an entanglement value of 0 corresponds with no entanglement). A) Dendrograms constructed from ground truth DNA-based single-nucleotide variant calls (DENDRO) and direct transcripts (hierarchical clustering) and compared by tanglegram. Colours correspond to individual cell lines (yellow: SKBR3, green: HCC827, and light blue: MCF7). Entanglement of the phylograms was 0.16 (an entanglement value of 1 corresponds with full entanglement of two phylograms, whereas an entanglement value of 0 corresponds with no entanglement).
(TIF)
