# Supplementary figures and images for: Clonal phylogenies inferred from bulk, single cell, and spatial transcriptomic analysis of epithelial cancers
Source: PLoS One. 2025 Jan 3;20(1):e0316475. doi: 10.1371/journal.pone.0316475 (PMC11698422; doi:10.1371/journal.pone.0316475)

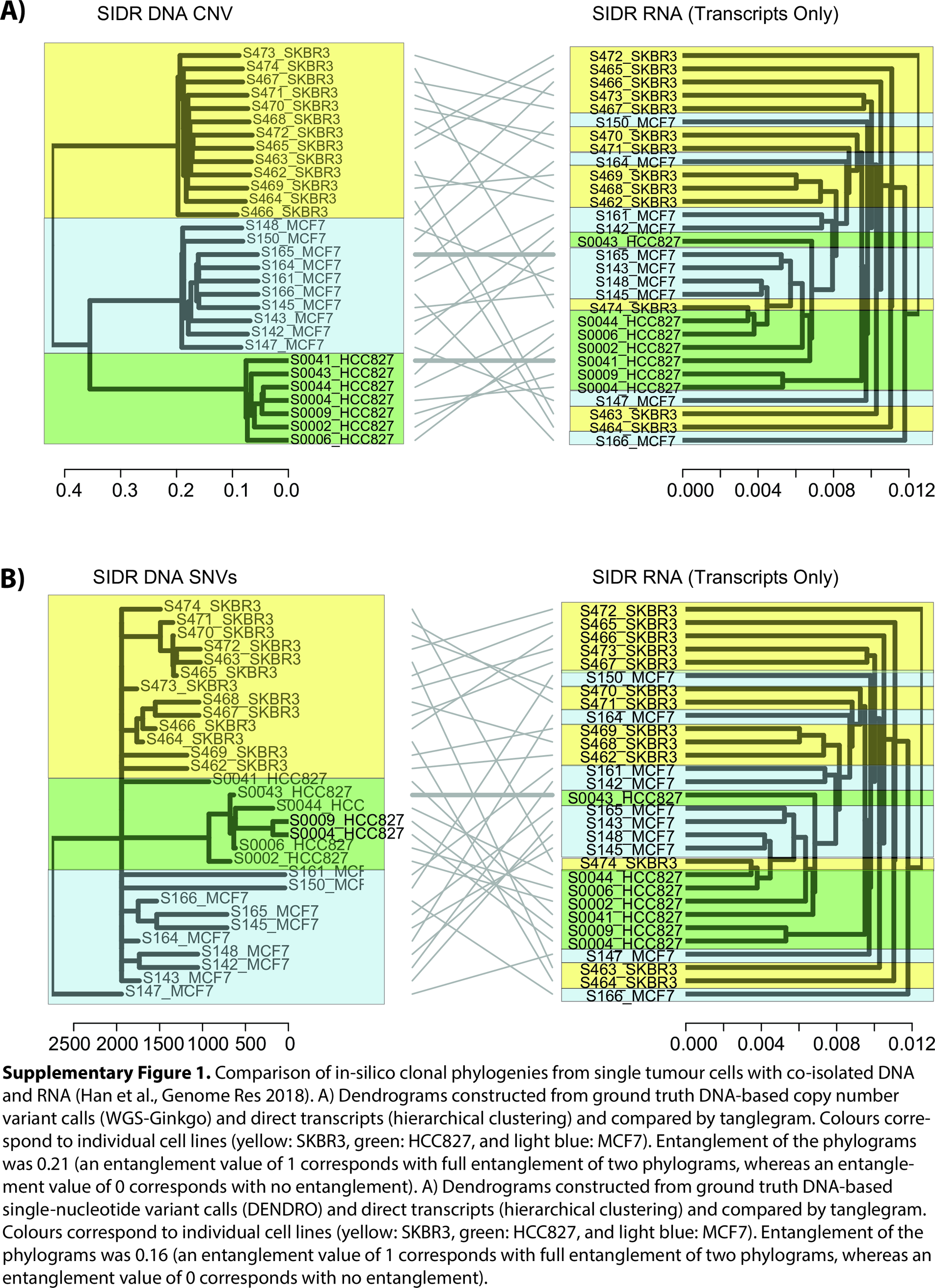

Supplement: S1 Fig — A) Dendrograms constructed from ground truth DNA-based copy number variant calls (WGS-Ginkgo) and direct transcripts (hierarchical clustering) and compared by tanglegram. Colours correspond to individual cell lines (yellow: SKBR3, green: HCC827, and light blue: MCF7). Entanglement of the phylograms was 0.21 (an entanglement value of 1 corresponds with full entanglement of two phylograms, whereas an entanglement value of 0 corresponds with no entanglement). A) Dendrograms constructed from ground truth DNA-based single-nucleotide variant calls (DENDRO) and direct transcripts (hierarchical clustering) and compared by tanglegram. Colours correspond to individual cell lines (yellow: SKBR3, green: HCC827, and light blue: MCF7). Entanglement of the phylograms was 0.16 (an entanglement value of 1 corresponds with full entanglement of two phylograms, whereas an entanglement value of 0 corresponds with no entanglement). (TIF) [file pone.0316475.s001.tif]
